# Investigation of Solar Waste Sand as a Supplementary Raw Material for Ordinary Portland Cement Clinker Production

**DOI:** 10.3390/ma19153336

**Published:** 2026-08-05

**Authors:** Robyn Erika Smith, Paramespri Naidoo, Adewumi John Babafemi, Guven Akdogan

**Affiliations:** 1 Department of Chemical Engineering, Stellenbosch University, Private Bag X1, Stellenbosch 7599, South Africa; robynerikasmith.res@gmail.com (R.E.S.); prathiekan@sun.ac.za (P.N.); gakdogan@sun.ac.za (G.A.); 2 Department of Civil Engineering, Stellenbosch University, Private Bag X1, Stellenbosch 7599, South Africa; 3 Department of Mining and Mineral Engineering, Institute of Northern Engineering, College of Engineering and Mines, University of Alaska Fairbanks, Fairbanks, AK 99775-580, USA

**Keywords:** solar waste sand, clinker, cement, electronic waste, photovoltaic (PV) panels

## Abstract

This investigation evaluated the suitability of solar waste sand (SWS), derived from recycled photovoltaic panels, as a raw material in clinker production. Raw materials were prepared, analysed, and used to design reference and SWS raw mix kneaded balls. The balls were subjected to sintering tests with varying peak temperatures; the clinker mineralogy was analysed using X-ray diffraction (XRD). The SWS raw mix contained 13.14% SWS with 78.31% and 8.55% of limestone and clay, respectively. The SWS clinker had 55.64 ± 2.26% C_3_S, 18.67 ± 1.27% β-C_2_S, 1.57 ± 0.35% CaO_f_, and 3.70 ± 1.41% MgO_f_ at 1350 °C, all within recommended ranges. Minor oxides, including Na_2_O, K_2_O, and MgO, reduced the required sintering temperature relative to the typical 1450 °C, as expected. The presence of an amorphous layer suggested the optimal temperature may be around 1350 °C. Furthermore, the high Na_2_O content in the SWS clinker is believed to have helped stabilise β-C_2_S. Low C_4_AF and the absence of C_3_A might indicate its suitability as a clinker for sulfate-resistant cements. Incorporating SWS directly into the raw mix for clinker production is an innovative approach to both diverting solar waste from landfills and partially supplementing sand and limestone.

## 1. Introduction

The cement industry plays a crucial role in global infrastructure development; however, it is also a major contributor to carbon emissions. In recent years, the industry’s emissions have accounted for approximately 8% of global anthropogenic CO_2_ emissions [1]. Approximately 40% of these emissions are due to the combustion of coal to provide the necessary energy for clinker production, while the other 60% is released due to the calcination reactions occurring during the clinkering process [2]. Ordinary Portland cement (OPC) manufacturing generates approximately 985 kg CO_2_ per ton of cement [1]; however, this varies depending on the raw materials used. Various strategies to lower emissions have been employed in industry, including the use of alternative fuels and supplementary cementitious materials (SCMs) such as fly ash, slag, calcined clays, limestone, and other alternative raw materials in the kiln feed used in cement mixtures [3] to reduce cement’s high carbon footprint associated with limestone calcination.

Costa and Ribeiro [4] reported that the overall CO_2_ production decreased by 4.90% when incorporating construction waste. Although no studies have reported the use of SWS as a raw material in clinker production, the similarity in its chemical composition to waste glass suggests it could be a viable candidate for incorporation into the raw mix.

The raw mix typically comprises limestone, clay, and sand, which provide the key oxides for clinker production: CaCO_3_ (which decomposes to CaO), SiO_2_, Al_2_O_3_, and Fe_2_O_3_ [1]. If the proportion of one of the major oxides in the raw mix is insufficient, iron ore (source of Fe_2_O_3_), bauxite (source of Al_2_O_3_), and sand (source of SiO_2_) are often added to the raw mix to correct the oxide compositions [5,6,7].

CaCO_3_ and SiO_2_ are predominantly used to form the primary clinker phases (C_3_S, C_2_S, C_3_A, and C_4_AF), while Al_2_O_3_ and Fe_2_O_3_ act as fluxing agents that enhance burnability by reducing diffusion limitations [8].

Raw mix proportioning is important to ensure that the necessary component concentrations are present to obtain the desired clinker quality [9]. The Lime Saturation Factor (LSF), Silica Modulus (SM), and Alumina Modulus (AM) are important parameters used to determine the optimal raw mix make-up [8]. The Lime Saturation Factor (LSF) provides an indication of the amount of CaO available to react with the other main oxides within the feed [4]. This is an important consideration as it determines the degree of burnability within the kiln, which is negatively influenced by the presence of unreacted CaO, also known as free lime or CaO_f_ [4]. To mitigate the risk of CaO_f_, LSF should be kept below the value of one. In addition to feed control, the LSF parameter dictates the ratio of alite (C_3_S) to belite (C_2_S) formation in the clinker [10]. LSF should be chosen to ensure sufficient quantities of both clinker phases are present in the final product, because C_3_S promotes early strength while C_2_S ensures long-term strength in cement pastes [8]. For OPC clinker, the optimal LSF falls within the 0.92 to 0.98 range [4,10,11]. The Silica Modulus (SM) is associated with raw mix burnability, the occurrence of burning zone coating, and ring formation in the kiln. Lower SM values are often linked to increased burnability and burning zone coating [10], which promotes higher conversion rates and protects the refractory lining of the kiln from chemical attack. For OPC, the optimal SM is between 2 and 3 [4,10,11]. The Alumina Modulus (AM) is also associated with the burnability of the raw mix. It indicates the relative proportions of Al_2_O_3_ to Fe_2_O_3_ in the raw mix and, consequently, the tricalcium calcium aluminate (C_3_A) to ferrite (C_4_AF) ratio in the clinker [12]. The main purpose of Al_2_O_3_ and Fe_2_O_3_ is to form a molten flux within the kiln, which facilitates the clinkering reactions [13]. The optimal AM is typically around 1.6; however, this value may vary slightly in the presence of minor oxides such as Na_2_O to maintain an appropriate melt viscosity [4,10,11].

The empirical correlations and the typical ranges for these parameters used in OPC raw mixture proportioning are reported in Table 1 [4,10,11].

The four major clinker phases in OPC clinker are belite (larnite, β-C_2_S), alite (hatrurite; C_3_S), calcium aluminate (C_3_A), and ferrite (brownmillerite; C_4_AF), with C_3_S and C_2_S being classified as silicate phases and C_3_A and C_4_AF as matrix phases. It is widely known that the reaction mechanisms and kinetics for clinker formation are complex, with multiple factors influencing the quality of the product formed. These factors include raw mix properties (e.g., oxide composition and mineralogy), particle size, homogeneity, and the process conditions (e.g., temperature, holding times, cooling rate) [6]. Four major reaction steps occur during clinkering; these include (1) limestone decarbonation, (2) solid–solid state reactions, (3) liquid–solid sintering reactions, and (4) clinker microstructure reorganisation during cooling [14]. The recommended heating cycle for laboratory clinker production is heating at a controlled rate of 5 °C/min to 900 °C and holding for 30 min to ensure complete decarbonation. Subsequently, the temperature is increased at the same rate to the peak value, where the samples are maintained for an additional 30 min before being rapidly cooled to ambient temperature [4,7,15]. Important factors within the cycle include the holding times, peak temperature, and cooling rate. After the sintering process, it is important to air cool from the peak temperature to the ambient conditions within a short period of time, as recommended by Zhao et al. [7], Costa and Ribeiro [4], Tsakiridis et al. [15], Badanoiu et al. [3] and Telschow [6].

Industrial waste often contains the essential oxides required for clinker formation, and waste products are increasingly used as viable raw material substitutes for clinker production. Steel slag [15], construction waste [4], and waste glass [3,16] are examples of wastes that have partially substituted limestone, clay or sand in the raw mix. Coal ash [17], copper tailings [18], and contaminated waste glass [19] have all been successfully incorporated to produce cement mixtures with suitable chemical and mechanical characteristics. Recently, Zele et al. [20] and Bastiaanse [21] demonstrated that solar panel glass waste sand (SWS)—obtained from crushed end-of-life (EOL) photovoltaic (PV) solar panels—could be a viable SCM. The significance of the work by Zele et al. [20] lies in demonstrating that the use of solar panel waste in cement production can simultaneously offer possible carbon reduction opportunities and provide a practical solution for solar waste management. There has been an increased uptake of solar as a renewable energy source, with PV panel waste projected to reach 8 million tons by 2030, and 78 million tons by 2050 [22]. South Africa is predicted to account for 80,000 tons of the total global PV panel waste in 2030 and 1,000,000 tons by 2050 [23]. Therefore, upcycling EOL solar panels, such as the use of solar waste sand (SWS) in Portland cement production, is necessary.

This study evaluates the feasibility of replacing conventional raw materials with solar waste sand in Portland clinker production by examining how sintering peak temperature influences phase formation. No previous studies have reported the use of SWS as a raw material in clinker production. The mixture for the raw meal was designed based on the analyses of the raw materials, and within the typical OPC range, producing a reference and solar waste sand clinker with SWS substituting part of the limestone and clay. The clinker balls were sintered at varying peak temperatures, and the clinker mineralogy was analysed. Electronic waste such as solar waste sand contains essential oxides required for clinker formation; therefore, it serves as a viable raw material substitute for clinker production.

## 2. Materials and Methods

### 2.1. Sample Preparation

**Figure 1:**
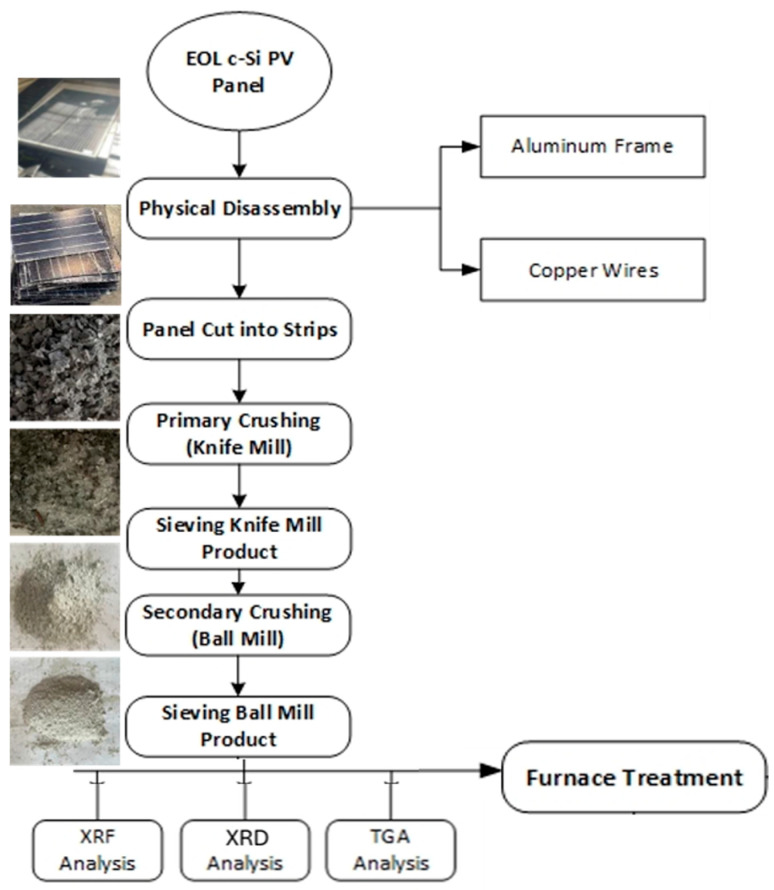
Generalised flow diagram for sample preparation of SWS.

The materials, Kulubrite 5 limestone (Idwala Pty Ltd), Kaolinite clay (Durbanville quarry) and silica sand (Malmesbury), were sourced locally from the Western Cape region, South Africa. EOL c-Si PV panels were used as SWS with a particle size below 75 µm, which was prepared by dismantling, knife-ball milling, and sieving (Figure 1). The ball mill used was manufactured by Rhologan Engineering, Pty Ltd., South Africa and the knife mill was manufactured by ABC Hansen Africa, South Africa.

Ferric oxide powder (analytical grade, Sigma-Aldrich 98% purity, St. Louis, MO, USA) and distilled water were acquired from the department laboratories. XRF analysis was performed on the feed materials. The PANalytical Axios Wavelength Dispersive spectrometer at the Central Analytical Facilities at Stellenbosch University, Cape Town, South Africa, was used. The instrument is fitted with a Rh tube and the following analysing crystals: LIF200, LIF220, PE 002, Ge 111 and PX1. The raw intensities were measured with the SuperQ PANanalytical software 6, maintained and licensed through Malvern Panalytical.

### 2.2. Sample Characterisation

The mineralogical and phase compositions of the various samples were determined using XRD in conjunction with the Rietveld method. The X-ray diffractograms were obtained using an Aeris diffractometer (MALVERN Pananalytical, Worcester, UK) equipped with a PIXcel detector and fixed slits, using Fe-filtered Co-Kα radiation. The spectrum was obtained in the 2θ angle range of 0–80°. The clinker phases were identified using X’Pert HighScore Plus software https://www.cambridge.org/core/journals/powder-diffraction/article/highscore-suite/C5F7C0DD3DA96EA5E0CFF1A0C9C87DF3 (accessed on 4 July 2026), MALVERN, Panalytical, Worcester, UK [24]. The amorphous content was determined by the External Standard Method (k-factor).

### 2.3. Sintering Experiments

Table 2 summarises the raw material proportions of the two sample types considered in experimental work. A SWS mix of two size classes—class 1 (d < 50 µm) and class 2 (50 < d < 75 µm) was used for this investigation. The raw materials were combined according to the proportions in Table 2 to produce the reference and SWS raw mix. For the SWS raw mix, LSF, SM, and AM of 0.98, 2.5, and 1.6 were achieved using an Excel Solver. The raw mixes were mixed at a water-to-solids ratio of 0.28 and moulded into balls with a diameter of 10 mm. The raw mix balls were placed into crucibles and dried in the Memmert UN30 electric oven at 100 °C for 12 h to remove the free moisture.

**Figure 2:**
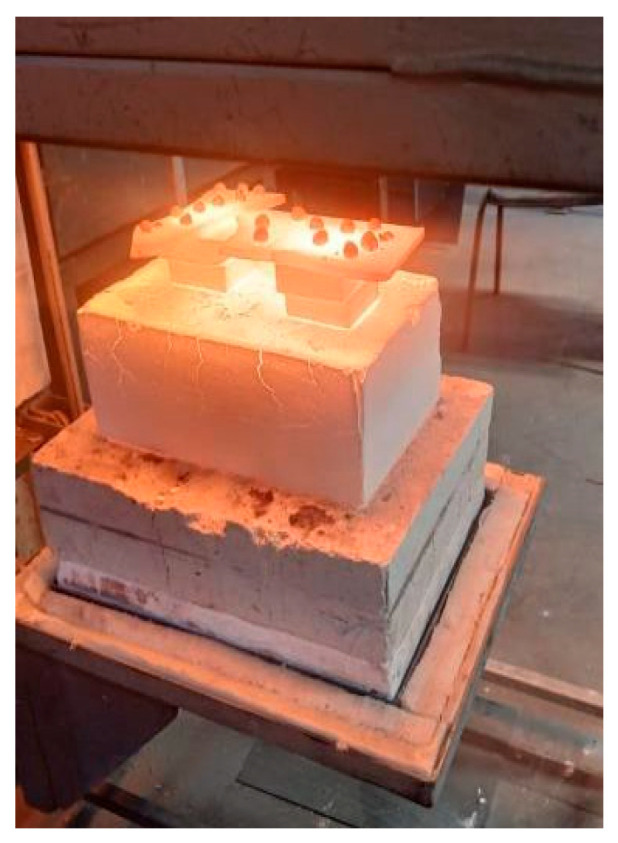
A drop-down oven base ensured rapid air cooling of the samples.

The sintering tests were conducted using a 12 kW bottom-loading chamber furnace at Thermopower (SA), in a furnace manufactured by Thermopower Furnaces SA (Pty) Ltd. The unit is heated with molybdenum silicide electric heating elements. The drop-down furnace assembly provided rapid air cooling (Figure 2). Experiments were conducted initially at 1250 °C, 1350 °C, and 1450 °C, and 1300 °C was added at a later stage.

The test conditions are given in Table 3. The samples were placed in the furnace, heated to 900 °C at a maximum rate of 5 °C/min and held at this temperature for a time interval. The spheres were then heated to the specified peak temperature and kept at this temperature for the peak holding time, as specified in Table 3. After the hold, the furnace floor was lowered out of furnace to allow rapid air cooling of the samples.

## 3. Results and Discussion

### 3.1. Characterisation of Feed Materials

**Figure 3:**
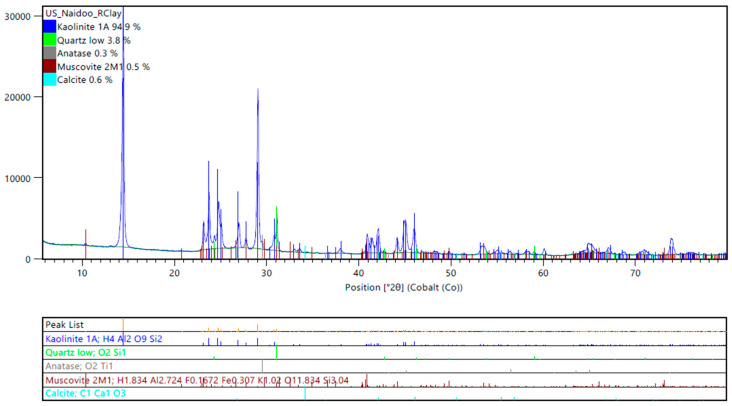
XRD pattern and quantitative analysis of the clay.

**Figure 4:**
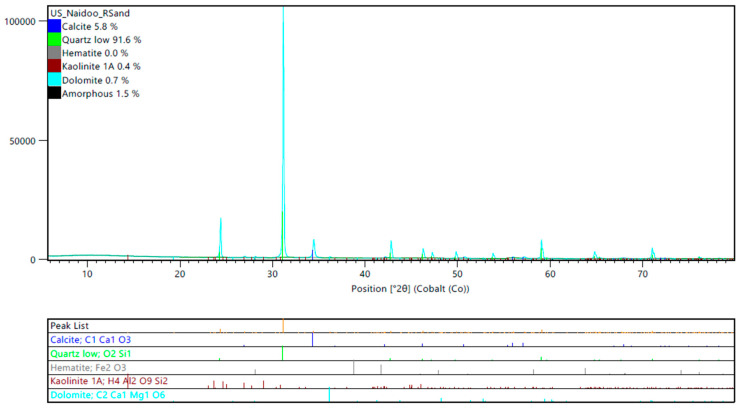
XRD pattern and quantitative analysis for the sand.

**Figure 5:**
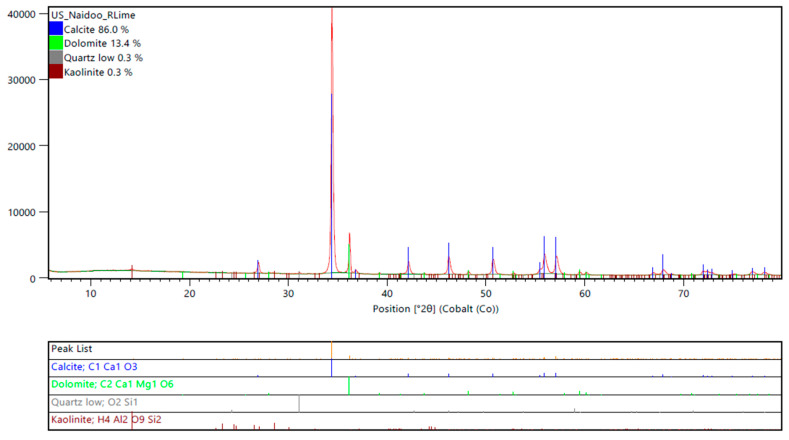
XRD pattern and quantitative analysis for the limestone. (red color: Calcite peak intensity, artifact left from measurement)

Table 4 presents the compositions of the materials used as reported by XRF analysis with the loss of ignition (LOI), with the XRD patterns and quantitative information provided in Figure 3, Figure 4 and Figure 5.

From the XRD pattern and quantitative information in Figure 3, it can be seen that the clay used in this investigation contained 94.9% Kaolinite (Al_2_(Si_2_O_5_)(OH)_4_), with 3.8% Quartz (SiO_2_), 0.3% Anatase (TiO_2_), 0.5% Muscovite (KFe_0.307_Al_2.724_Si_3.04_O_10_(OH)_1.834_), and 0.6% Calcite (CaCO_3_). Compared to the literature, there are limited Fe_2_O_3_ sources compared to Al_2_O_3_ in the clay. The XRD results support the XRF findings in Table 4, showing that the clay contains predominantly Al_2_O (37.52%), with limited Fe_2_O_3_ (0.42%). Examining Table 2 shows that none of the raw materials contained sufficient Fe_2_O_3_. Therefore, the use of only clay and limestone was insufficient to achieve well-designed raw mixes with acceptable LSF, AM, and SM values, as stipulated in Table 1.

In industrial clinker production, corrective materials such as iron ore (Fe_2_O_3_ source), bauxite (Al_2_O_3_ source), and sand (SiO_2_ source) are commonly added to balance the oxide composition [5]. Therefore, additional pure Fe_2_O_3_ had to be added to the reference and SWS clinker raw mixes. The silica sand was relatively pure, containing 98.12% SiO_2_ according to the XRF results in Table 4. The XRD pattern and quantitative information are presented in Figure 4. It can be seen that the SiO_2_ in the sand is 91.6% Quartz (SiO_2_).

The results for limestone depicted in Figure 5 are predominantly comprising 86.0% calcite (CaCO_3_), with 13.4% dolomite. There are traces of kaolinite and quartz in the limestone, but the quantities are negligible. The XRD results further confirm that the limestone is well suited for clinker production, as its high CaCO_3_ content ensures an adequate source of CaO for clinker phase formation.

**Figure 6:**
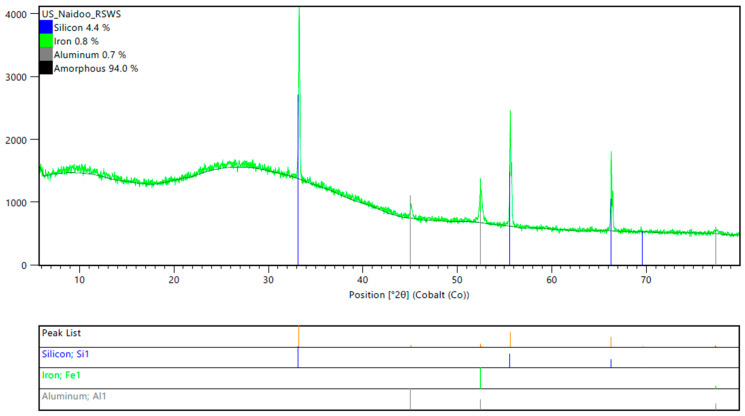
XRD pattern and quantitative analysis for the SWS.

From Table 5, it is evident that SWS had a high SiO_2_ content (73.57 ± 0.53%). This indicates that SWS could serve as a complete replacement for sand in the raw clinker mix. The XRD pattern and quantitative analysis of SWS are presented in Figure 6. The SWS consisted of approximately 94% amorphous material, most likely glass (SiO_2_), supporting its suitability as a sand substitute. This is consistent with the approach described by Badanoiu et al. [3], in which waste glass was used to replace sand, with the SiO_2_ contents only differing by 4.61%, indicating that the two are closely comparable.

Furthermore, SWS also contained 7.73 ± 0.31% CaO, which highlights that a portion of the limestone could be replaced by SWS, mitigating emissions related to limestone decarbonation. There were also substantial amounts of 10.89 ± 0.44% Na_2_O, which could negatively affect clinkering and the subsequent cement properties due to ASR reactions. In contrast, some alkali content could be beneficial during clinker cooling by stabilising β-C_2_S and preventing the unwanted γ-C_2_S formation. It could also lower the eutectic temperature of the CaO-SiO_2_-Al_2_O_3_-Fe_2_O_3_ quaternary system, resulting in improved raw mix burnability and lower energy consumption. Gies and Knöfel [25] argued that fast cooling (e.g., using a grate cooler) helps lock in the high-temperature crystalline structures of alite and belite. It is highly advantageous because it restricts the growth of large aluminate and ferrite crystals, resulting in a more hydraulically reactive clinker that is easier to grind. The reaction of alkalis with sulfur (forming K_2_SO_4_ and Na_2_SO_4_) typically keeps the alkalis as separate phases between the main clinker crystals. If sulfur is deficient, the alkalis are forced to incorporate directly into the crystal lattices of belite or the aluminate phase (C_3_A). The incorporation of alkalis into the belite lattice stabilises highly reactive high-temperature forms of the mineral (like α and α’ belite), significantly increasing the 28-day compressive strength of the cement. Gobbo et al. [26] also reported that as clinker cools from the burning zone, the remaining liquid phase crystallises into aluminate and ferrite phases. The presence of alkalis alters the viscosity and temperature at which this melt solidifies, often stabilising high-temperature and orthorhombic crystalline modifications instead of their stable, low-alkali forms.

Based on the raw mix design to maintain the LSF, SM and AM targets of 0.98, 2.50 and 1.60, respectively, a maximum of 13.14% SWS was used. The limestone content in the SWS relative to the reference raw mix decreased by 1.22%, the clay by 5.84%, and the sand was completely replaced by SWS. The SWS content was more than double the 5.59% maximum waste glass content in the clinker raw mix prepared by Badanoiu et al. [3]. Although both studies used comparable LSF, SM and AM values and the two materials (waste glass and SWS) were found to be compositionally similar, the difference can be attributed to the oxide compositions of limestone and clay in this study, which allowed for a greater incorporation of SWS into the raw mix. Previous studies reported the use and incorporation of construction waste at 14.29% [4], steel slag at 10.5% [15] and fly ash leaching residue at 26.98% [7]. The percentage inclusion of SWS content, i.e., at 13.14%, lies within the ranges for waste addition to clinker raw mixes. However, the potential influence of higher Na_2_O levels should be monitored and further investigated.

### 3.2. Sintering Tests

#### 3.2.1. Sintering 1 at 1450 °C: Reference Samples

**Figure 7:**
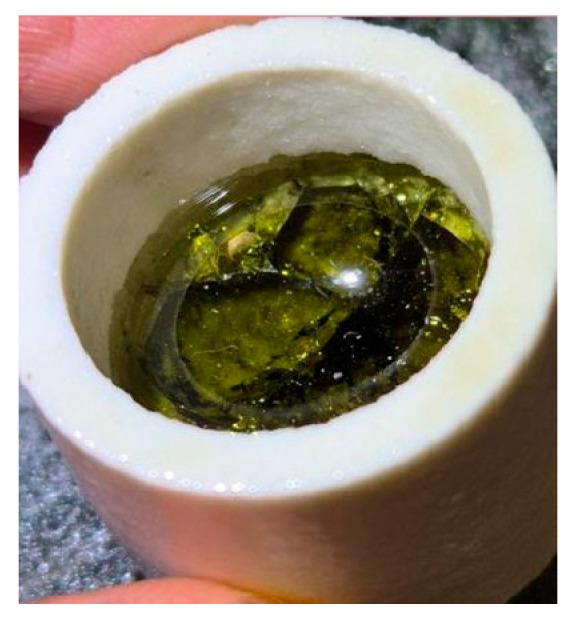
Amorphous product formed during Sintering 1 (sample type: reference; sample holder: ceramic crucible; peak temperature: 1450 °C).

Sintering 1 was set at 1450 °C to test the method and establish whether the sintering and holding time was adequate. The thermal treatment during Sintering 1 did not result in a uniformly sintered product as desired. The product predominantly comprised a green glass with the presence of small quantities of sintered product below the glass surface, as seen in Figure 7.

There are multiple possible explanations for the glass product that formed instead of sintered clinker. When examining Figure 7, there has been clear corrosion of the crucible where it is in contact with the material. This is an indication that the ceramic crucibles used reacted with the samples during Sintering 1. Ceramic crucibles were selected due to this material also being used in the study by Costa and Ribeiro [4], ensuring consistency and comparability with previous research. However, these crucibles are primarily composed of alumina (Al_2_O_3_), which is one of the four major oxides responsible for clinker formation.

Since Al_2_O_3_ actively participates in the formation of the clinker phases, its presence as part of the crucible material introduced an uncontrolled variable. In homogeneous mixtures, the solid-state reactions occur predominantly below 1250 °C [6], making the interaction between crucible-derived Al_2_O_3_ and the raw mix particularly relevant to the clinkering process. This additional source would have significantly increased the effective Al_2_O_3_ participating in the reactions, thereby causing the Alumina Modulus (AM) to deviate from the intended raw mix design value. The raw mix was designed with an AM of 1.6, which is reported in the literature as the theoretical optimum for flux formation and burnability [3,10,12]. However, the uncontrolled introduction of crucible-derived Al_2_O_3_ could have prevented stable control of the clinkering process. Costa et al. [12] reported that excessive AM values (i.e., above the optimal range) increase both the volume and viscosity of the melt/flux. This, in turn, reduces clinker burnability by delaying silicate conversion and C_3_S formation [12], which may explain the observed lack of sintering. Consequently, for the remaining sintering tests, the samples were transferred to recrystallized silicon carbide to eliminate crucible interference and preserve the integrity of subsequent results.

#### 3.2.2. Sintering Tests 2 to 4: Reference and SWS Samples

**Figure 8:**
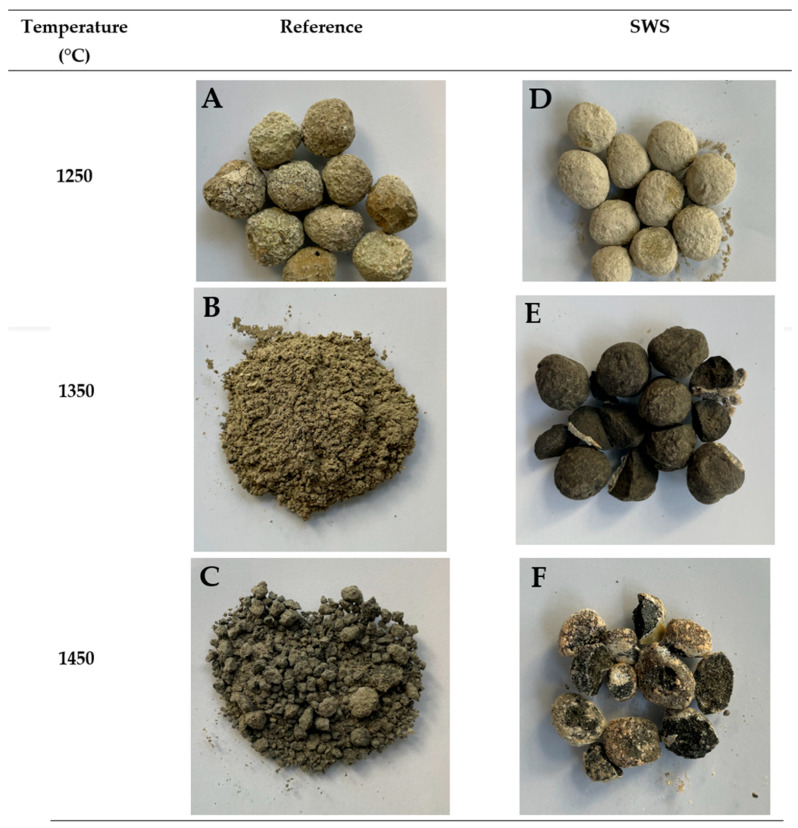
(**A**–**F**) Reference and SWS clinker appearance at different peak sintering temperatures.

The macroscopic appearance of the reference and SWS clinker at the different peak temperatures investigated is illustrated in Figure 8A–F.

##### Sintering 2 with Peak Temperature at 1250 °C

Both reference and SWS samples were heated in the same sintering test. From Figure 8A, it can be seen that the reference clinker from Sintering 2 retained a spherical form, with a colour change to light brown-yellow. Similarly, the SWS clinker (Figure 8D) also displayed a light brown-yellow colour, which is indicative of C_2_S formation, accompanied by C_3_A and C_4_AF. These macroscopic features are consistent with the visual observations reported by Zhao et al. [7] for the fly ash leaching residue clinker produced at a peak temperature of 1250 °C. However, the light colour of both clinkers suggests under-burning, where the characteristic OPC clinker phases were not formed in the correct proportions. Comparable under-burning appearances were also noted by Du Toit [14] for clinker produced at an inadequate peak temperature. Both the reference and SWS clinkers were easily crushed, which may be attributed to the absence of C_3_S. During quenching, increased oxidation occurs. However, in the absence of C_3_S, limited bonding and fusion take place between clinker phases, resulting in friable nodules, therefore explaining their ease of crushing.

##### Sintering 3 with Peak Temperature at 1350 °C

Both reference and SWS samples were heated in the same sintering test. The reference clinker completely disintegrated after sintering, as shown in Figure 8B. Although the nodules retained their structural integrity during thermal treatment, they disintegrated upon cooling. This clinker dusting was caused by the majority of β-C_2_S transforming into γ-C_2_S during cooling, which was likely caused by an insufficiently rapid cooling rate. The resulting volume expansion led to clinker dusting and loss of cohesion. Similar behaviour was reported by Zhao et al. [7] for clinkers produced at 1350 °C. The light brown colour of the reference clinker indicates that C_3_S formation had increased relative to the 1250 °C clinker, although complete phase development could not have been achieved yet. The colour remained lighter than that of typical industrial clinkers, which are usually dark grey or black [14]. The nodules exhibited increased hardness during grinding, with the presence of dense, black C_3_S-rich particulates suggesting partial sintering.

In contrast, the SWS clinker (Figure 8E) produced at 1350 °C retained its form during sintering and cooling, exhibiting a final dark grey colour indicative of high C_3_S content. The clinker nodules were hard and difficult to crush, confirming the formation of well-sintered C_3_S. There was no clinker dusting or crumbling, indicating that β-C_2_S was effectively stabilised during cooling. Zhao [7] reported that the presence of alkalis Na_2_O and K_2_O at concentrations above 1.2% and 1.5% stabilised β-C_2_S. According to the SWS raw mix design, the Na_2_O content was 2.02%, which could have stabilised the β-C_2_S, with limited K_2_O being detected. In contrast, the reference raw mix contained only 0.01% Na_2_O. The disparity in Na_2_O content may explain why the reference clinker crumbled while the SWS clinker nodules retained their form under the same cooling conditions. Further macroscopic examination of Figure 8E revealed the presence of an amorphous layer at the base of each clinker nodule that did not reincorporate during cooling. This suggests that excessive melt formation occurred at 1350 °C, resulting in an amorphous phase during solidification.

The eutectic temperature of the CaO-SiO_2_-Al_2_O_3_-Fe_2_O_3_ system indicates liquid formation, and temperatures above this increase the liquid fraction, thereby promoting C_3_S formation. The eutectic temperature of the quaternary system varies depending on the presence of other oxides (including Na_2_O and MgO) in the raw mix [14]. However, exceeding the eutectic temperature by too great a margin can result in excessive melt formation that cannot reincorporate during cooling. This observation indicates that 1350 °C may be slightly above the optimal peak temperature for SWS clinker formation, where an ideal balance of liquid phase promotes C_3_S formation and proper phase integration upon cooling. As previously discussed, the SWS clinker fired at 1300 °C appeared under-burnt, suggesting that the optimal sintering temperature likely lies between 1300 °C and 1350 °C, but closer to 1350 °C.

##### Sintering 4 with Peak Temperature at 1450 °C

Both reference and SWS samples were heated in the same sintering test. The reference clinker produced at a peak temperature of 1450 °C is shown in Figure 8C. These nodules retained their shape during sintering but disintegrated during cooling. This behaviour is attributed to volume expansion and subsequent clinker dusting caused by β-C_2_S to γ-C_2_S transformation, which was also observed for the reference clinker at 1350 °C. Once again, the difference in alkali content between the reference and SWS clinker likely contributed to the contrasting behaviour, with the reference clinker nodules crumbling while the SWS clinker nodules retained their form under the same cooling conditions. In addition, the nodules were dark grey in colour and hard to grind, indicating C_3_S formation.

The SWS clinker produced at the same peak temperature (1450 °C) consisted of three different products: an amorphous yellow crust, a hard, solid black nodule, and a green-grey powder surrounding the nodule. The dark grey nodule (Figure 8F) has the typical appearance of clinker, with the dark colour indicating adequate sintering and substantial C_3_S. The large yellow rings at the base of each nodule resembled kiln rings commonly observed in industrial manufacturing, which are often associated with operational issues. The amorphous rings were more pronounced than in the SWS clinker fired at 1350 °C, suggesting that 1450 °C may be well above the optimal peak temperature for SWS clinker formation. At this temperature, excessive melt formation occurred, exceeding the ideal amount needed for C_3_S formation and proper phase integration upon cooling. Zhao et al. [7] reported that the clinker produced at 1500 °C melted due to excessive liquid formation, mirroring the behaviour observed in this study.

##### Sintering 5 with Peak Temperature at 1300 °C with SWS

**Figure 9:**
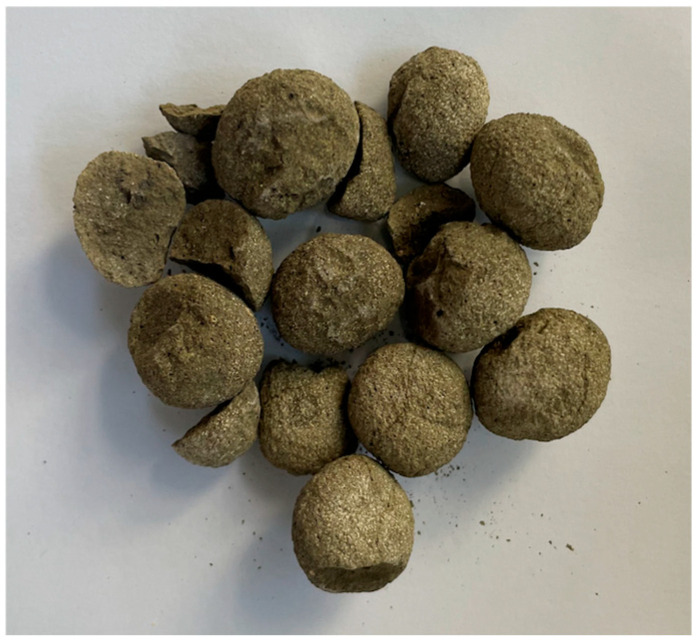
SWS clinker appearance at 1300 °C peak sintering temperature.

The SWS clinker nodules were fired at 1300 °C to assess the properties in the intermediate temperature range of 1250 and 1350 °C. As shown in Figure 9, the nodules retained their form during sintering and cooling and exhibited a brown colour, attributed to the high C_2_S content. Numerous dark grey spots were observed on the clinker surface, suggesting that progressively more C_3_S had begun forming at a peak sintering temperature of 1300 °C compared to 1250 °C. Zhao et al. [7] reported similar macroscopic observations at 1300 °C. Small amounts of material detached from the main bodies of the nodules, forming a grey powder at their bases. This dusting may have resulted from minor β-C_2_S to γ-C_2_S transformation during cooling, combined with the effect of relatively low C_3_S. content, which reduced fusion and bonding and rendered the nodules more friable. The colour was still lighter than typical industrial clinkers, which are usually dark grey or black [14].

### 3.3. Peak Temperature XRD Trends

**Figure 10:**
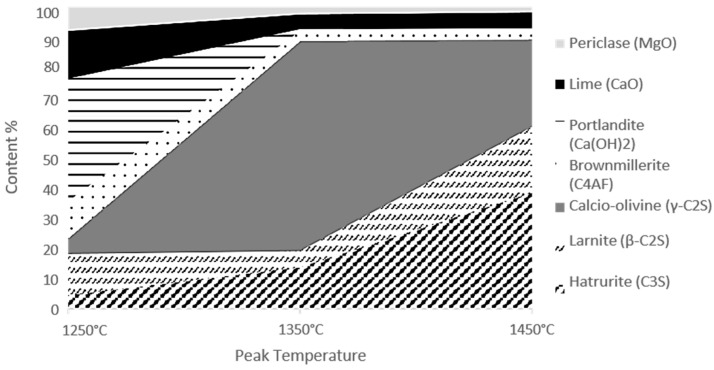
Normalised Reference clinker main XRD phase composition for the peak temperatures.

**Figure 11:**
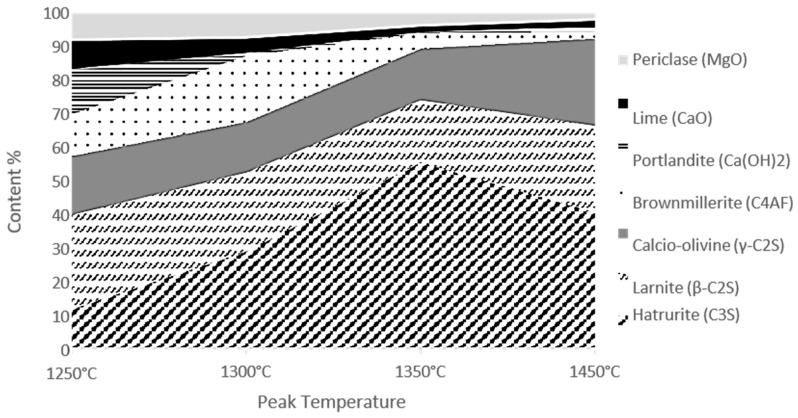
Normalised SWS clinker main XRD phase composition for the peak temperatures.

The XRD patterns for the major clinker phases present in the reference and SWS clinker are illustrated in Figure 10 and Figure 11, respectively. The values for the compositions at the different peak temperatures are reported in Table 6 and Table 7.

#### 3.3.1. Reference Clinker

Table 6 and Figure 10 provide the reference clinker main normalised XRD phase composition for the investigated peak temperatures. It can be seen that the alite (hatrurite; C_3_S) content increased with increasing peak temperature, reaching a maximum of 38.54% at 1450 °C. This aligns with the macroscopic colour changes observed, where the clinker darkened with increasing peak temperature due to increased C_3_S formation. However, the 1450 °C C_3_S content remained below the 50 to 70% typically required in clinker for adequate early cement strength [27]. The beta belite polymorph (β-C_2_S; larnite) content decreased from 13.77% at 1250 °C to 5.45% at 1350°, then increased to 21.79% at 1450 °C. The β-C_2_S content at 1450 °C is therefore in the 15 to 30% range expected for OPC clinker. This is necessary for long-term strength development in cement [14]. However, significant amounts of the belite polymorph γ-C_2_S (calcio-olivine) were present in all the reference clinkers. This phase formed from the transformation of β-C_2_S during slow cooling, causing volume expansion and clinker disintegration. At 1350 °C and 1450 °C, γ-C_2_S constituted 92.74% and 57.19% of the total belite content, indicating extensive β-C_2_S transformation. This supports the macroscopic observations regarding the crumbled appearance of the clinker (refer to Figure 8B,C). Note that the larger clumps observed in the 1450 °C clinker compared to the powdered clinker at 1350 °C can be attributed to lower γ-C_2_S relative to β-C_2_S at the higher temperature. Furthermore, the cooling rate in this study could have been insufficient, likely contributing to the low C_3_S content relative to β-C_2_S for the 1350 °C and 1450 °C sintering. C_3_S is thermodynamically stable only above 1250 °C and decomposes into C_2_S and CaO at lower temperatures [6]. Rapid cooling to below 1250 °C is therefore essential to preserve C_3_S in its metastable form. The slower cooling in this study could have allowed for the partial decomposition of C_3_S to β-C_2_S and CaO. The β-C_2_S then further transformed to γ-C2S at around 500 °C as cooling progressed, resulting in low C_3_S, reduced β-C_2_S, and high γ-C_2_S contents.

The brownmillerite (C_4_AF) content decreased from 12.34% at 1250 °C to 2.93% at 1450 °C, with the C_4_AF content at 1450 °C being significantly lower than the ±10% typically found in industrial-grade OPC [26]. This suggests that there was insufficient Fe^3+^ available in the melt to combine with Ca^2+^ and Al^3+^ to form C_4_AF upon cooling [15], resulting in low C_4_AF content. Although additional Fe_2_O_3_ was introduced to the raw mix to compensate for low iron content in the raw materials, the amount added could not have been adequate to ensure sufficient C_4_AF formation. Furthermore, quantitative XRD analysis detected no aluminate phase (C_3_A) in any of the reference clinkers, even though a 5 to 10% C_3_A content is desirable for OPC clinker [26].

Segata et al. [28] found that the presence of MgO can promote the formation of C_4_AF at the expense of C_3_A. The reference raw mix in this study contained MgO, which may have led to the low C_3_A content. In addition, the absence of the C_3_A phase and low C_4_AF content could also be an indication that the Alumina Modulus (AM) of 1.6 used for the raw mix may not have been optimal. It is therefore recommended that the effect of varying AM during raw mix proportioning on the formation of C_3_A and C_4_AF be investigated.

The CaO_f_ content in the reference clinker decreased from 15.74% at 1250 °C to 5.37% at 1450 °C. According to EN 197-1, CaO_f_ should not exceed 2 wt% [29]. Optimising the raw mix composition with respect to various feed compositions to achieve an optimum LSF, SM, and AM is recommended to improve clinker phase formation and reduce free lime content.

Periclase (MgO_f_) was also detected in notable amounts, decreasing from 7.87% at 1250 °C to 1.63% at 1450 °C. This reduction with increasing peak temperature reflects greater incorporation of Mg^2+^ into the melt and clinker phases during recrystallization, rather than forming a distinct periclase phase. For peak temperatures of 1350 °C and 1450 °C, MgO_f_ remained below the 5% limit specified in EN 197-1 [29], indicating that there would be minimal risk of compromised cement hydration if these clinkers were to be used in cement pastes.

#### 3.3.2. SWS Clinker

Table 7 summarises the SWS clinker normalised main XRD phase composition for the SWS clinker at the different peak temperatures investigated. Table 7 and Figure 11 visually represent this information. From Table 7 and Figure 8, it can be seen that the C_3_S content in the SWS clinker increased steadily with temperature, reaching a maximum of 55.64% at 1350 °C, before decreasing at 1450 °C. This trend aligns with the macroscopic observations, where the clinker colour darkens with increasing temperature, indicative of greater C_3_S formation (Figure 8D–F). From a C_3_S standpoint, 1350 °C can therefore be considered the best peak temperature for SWS clinker production investigated. However, as previously discussed, the presence of a thin amorphous layer at the base of the 1350 °C clinker nodules suggests that the optimal sintering temperature lies slightly below 1350 °C. The 55.64% C_3_S composition also fell within the recommended 50 to 70% range for high-quality clinker [27].

The β-C_2_S content was highest at 1250 °C, which decreased steadily up to 1350 °C, and then increased again at 1450 °C. The decrease from 1250 °C to 1350 °C reflects the reaction of β-C_2_S with CaO to form C_3_S. The β-C_2_S content remained within the recommended 15 to 30% range for the SWS clinker at all peak temperatures. However, only at 1350 °C were both the C_3_S and β-C_2_S contents within acceptable limits, 55.64% and 18.67%, respectively, further supporting 1350 °C as being close to the optimal peak temperature for SWS clinker formation.

Although no clinker dusting occurred for the 1350 °C clinker (Figure 8F), γ-C_2_S accounted for 15.53% of the clinker, nearly equal to the 18.67% β-C_2_S content. The high C_3_S content aided in bonding and fusion between clinker phases, resulting in hard nodules that retained their structure. Furthermore, the 2.02% Na_2_O present in the SWS raw mix could have contributed to the stabilisation of β-C_2_S, preventing clinker dusting. As previously discussed, Zhao et al. [7] reported that concentrations above 1.2% and 1.5% stabilised β-C_2_S and hindered unwanted transformation. However, the high proportion of γ-C_2_S, an inert phase, could reduce the hydraulic reactivity of the clinker during cement hydration. To mitigate the β-C_2_S transformation to γ-C_2_S, the cooling rate should be increased. For future experimental work, it is recommended to implement forced convective air cooling rather than relying solely on natural cooling.

The C_4_AF content initially increased from 12.52% at 1250 °C to 19.70% at 1300 °C, indicating increased solid-state reaction. This was followed by a decrease from 4.62% at 1350 °C to 1.95% at 1450 °C. At the identified near-optimal 1350 °C, the C_4_AF was lower than the recommended ±10% typically found in industrial-grade OPC [26]. As discussed for the reference clinker, it suggests that there was insufficient Fe^3+^ available in the melt to combine with Ca^2+^ and Al^3+^ to form C_4_AF upon cooling [15], resulting in the low C_4_AF content observed. The absence of C_3_A in all the SWS clinkers could be attributed to the presence of MgO in the SWS raw mix. Segata et al. [28] found that MgO can promote the formation of C_4_AF at the expense of C_3_A. Furthermore, the presence of Na_2_O increases the possibility of Al_2_O_3_ incorporation into the C_2_S and C_3_S crystal lattices, causing a reduction in Al_2_O_3_ available for C_3_A formation [26]. Since the SWS introduced 2.02% Na_2_O to the raw mix, this could be a viable explanation. However, further investigation is required to verify these effects in the SWS clinker. The absence of the C_3_A phase and low C_4_AF content at 1350 °C could also be an indication that the AM of 1.6 used for the raw mix proportioning may not have been optimal. Furthermore, the CaO_f_ content decreased with increasing peak temperature, measured as 8.41%, 4.33%, 1.57%, and 1.79% at 1250 °C, 1300 °C, 1350 °C, and 1450 °C, respectively. At 1350 °C, CaO_f_ was below the EN 197-1 limit of 2 wt%, indicating adequate raw mix burnability. Similarly, the periclase (MgO_f_) content decreased from 8.04% at 1250 °C to 1.95% at 1450 °C, with intermediate values of 7.36% and 3.70% at 1300 °C and 1350 °C, respectively. The decrease in MgO_f_ could be due to increased liquid phase formation with increasing peak temperature, which promotes the incorporation of Mg^2+^ into the melt rather than forming a distinct periclase phase. At the sintering temperature of 1350 °C, the MgO content was below the 5% acceptable limit specified in EN 197-1 [29].

#### 3.3.3. Reference and SWS Clinker Comparison

From the macroscopic observations and XRD results, the best reference clinker was produced at 1450 °C. However, this temperature did not yield clinker of acceptable quality, as indicated by the high γ-C_2_S and CaO_f_ contents, coupled with insufficient C_3_S formation and the clinker disintegrating upon cooling. In contrast, the optimal peak temperature for the SWS clinker was determined to be 1350 °C, which resulted in adequate C_3_S and β-C_2_S formation, with limited CaO_f_ and MgO. The results indicate that the eutectic temperature of the CaO-SiO_2_-Al_2_O_3_-Fe_2_O_3_ system was effectively reduced, which decreased the required peak sintering temperature by at least 100 °C in the SWS clinker relative to the reference and the typical peak temperature of 1450 °C. This reduction could be attributed to the impurities—particularly Na_2_O and MgO—introduced by replacing silica sand with SWS. The reference clinker disintegrated during cooling as a result of extensive β-C_2_S transforming to γ-C_2_S, while the SWS clinker retained its form under identical conditions. This difference could be due to the stabilising effect of Na_2_O in the SWS raw mix, which inhibited the β- to γ-C_2_S transformation.

#### 3.3.4. Amorphous Content

The discussion in the previous sections excluded the amorphous phase. However, all the reference and SWS clinkers produced contained significant quantities of amorphous material, with the values at each peak temperature summarised in Table 8. Acceptable amorphous content in industrial-grade clinkers ranges from 2 to 21% [30]. However, laboratory clinkers have been found to have significant amorphous content, with Whitfield and Mitchell [31] having clinker with up to 31.2% amorphous content. Kang et al. [32] reported a similar magnitude of amorphous phase of 30.7% for their clinker test. The reference clinker amorphous content ranged from 13.3% to 37.6%, while the SWS clinker had contents ranging from 36.6% to 53.4%. It can also be seen that there was no correlation between the peak temperature and the amount of amorphous phase formed. Except for the 1350 °C reference clinker, all the other clinkers had amorphous content much higher than reported in the literature. The excessive amorphous phase content could negatively impact the cement performance during and after hydration.

As previously discussed, rapid cooling to below 1250 °C is necessary to preserve C_3_S in its metastable form. Melt recrystallisation typically begins at approximately 1250 °C, although this temperature can vary slightly depending on the influence of minor oxides on the eutectic temperature of the system. These two important phenomena occur in approximately the same temperature region. Therefore, the rapid cooling required for C_3_S stabilisation can result in significant melt undercooling, which inhibits the recrystallization of C_3_A and C_4_AF, thereby promoting amorphous phase formation [6,33]. In the experiments conducted in this study, the cooling was complete within 20–25 min from the peak temperatures to ambient conditions. This gives approximate cooling rates between 50 °C/min and 70 °C/min.

Costa and Ribeiro [4], Zhao et al. [7], and Badanoiu et al. [3] reported the necessity of rapid cooling to form good-quality clinkers, with no mention of a slow-cooling step preceding rapid cooling. Although the true role of the amorphous phase is still under discussion in the scientific literature, we recommend that the influence of cooling rate be further investigated to identify the conditions that provide the optimal balance between C_3_S preservation and minimisation of amorphous phase formation.

## 4. Conclusions

This investigation evaluated the potential of SWS as a raw material for OPC clinker production at temperature ranges between 1250 °C and 1450 °C. The following conclusions are drawn:

(1) The oxide composition of the SWS exhibited minimal variability irrespective of the panel source or the particle size effects, indicating that SWS could be a chemically consistent and reliable raw material. The high percentage of SiO_2_ and CaO in SWS confirmed its suitability to replace sand and partially substitute limestone in the raw mix, potentially reducing CO_2_ emissions related to limestone decarbonation.
(2) With an LSF, SM, and AM of 0.98, 2.50, and 0.60, respectively, for the SWS raw mix, it was determined that a maximum of 13.14% SWS could be substituted. The SWS replaced all the sand, 1.22% of the limestone, and 5.84% of the clay. At 13.14%, the SWS addition remained within realistic limits for waste incorporation into clinker raw mixes.
(3) Macroscopic and XRD analyses indicated that the SWS clinker showed improved burnability, with C_3_S (55.64%) and β-C_2_S (18.67%) within the recommended ranges at 1350 °C, indicating that 1350 °C was the optimal sintering temperature. Moreover, the SWS clinker retained the nodular form, likely due to the Na_2_O stabilising β-C_2_S. In contrast, reference clinker required a higher temperature, 1450 °C, producing only 38.54% C_3_S content, well below the required target of 50% for high-quality clinker. Furthermore, its elevated γ-C_2_S concentrations induced clinker degradation.

## Figures and Tables

**Table 1:** LSF, SM and AM values for OPC [4,10,11].

Parameter	Equations	Typical OPC Range
Lime Saturation Factor	$LSF=\frac{C}{2.80S+1.18A+0.65F}$	0.92–0.98
Silica Modulus	$SM=\frac{S}{A+F}$	2–3
Alumina Modulus	$AR=\frac{A}{F}$	1–4

C = CaO; S = SiO_2_; A = Al_2_O_3_; F = Fe_2_O_3_; all chemical components represent weight percentages.

**Table 2:** Raw meal compositions (wt%) for the reference and maximum SWS substitution samples (LSF = 0.98, SM = 2.5, AM = 1.6).

Sample	Limestone (%)	Clay (%)	Sand (%)	SWS (%)	Add. Fe_2_O_3_ (%)
Reference	79.28	9.08	9.57	0	2.07
SWS	78.31	8.55	0	13.14	1.97

**Table 3:** Conditions of sintering tests.

Parameter	Sintering 1	Sintering 2	Sintering 3	Sintering 4	Sintering 5
# of clinker balls	R: 30	R: 10	R: 10	R: 10	R: 10
	SWS: 0	SWS: 10	SWS: 10	SWS: 10	SWS: 10
Max. heating rate (°C/min)	5	5	5	5	5
Peak temperature (°C)	1450	1250	1350	1450	1300
Peak hold (min)	15	30	30	30	30

R—reference; SWS—solar waste sand.

**Table 4:** Main oxide compositions of the raw materials determined by XRF analysis.

Raw Material	Main Metal Oxide (wt%)							
	Al_2_O_3_	CaO	Fe_2_O_3_	K_2_O	MgO	Na_2_O	SiO_2_	LOI
Kaolinite clay	37.52	0.22	0.42	0.08	BDL	0.04	47.50	13.75
Silica sand	0.14	0.79	0.03	0.02	BDL	0.01	98.12	0.87
Kulubrite 5 limestone	0.02	54.92	0.05	BDL	1.25	BDL	0.32	43.06

BDL—Below detection level.

**Table 5:** Solar waste sand characterisation for different size classes.

Sample Type	Main Metal Oxide (wt%)							
	Al_2_O_3_	CaO	Fe_2_O_3_	K_2_O	MgO	Na_2_O	SiO_2_	LOI
SWS 1 (d < 50 µm)	1.84	7.86	0.76	0.32	3.47	11.19	72.87	1.77
SWS 2 (d < 50 µm)	1.59	7.97	0.54	0.34	3.43	11.29	73.69	1.14
SWS 3 (d < 50 µm)	1.63	7.93	0.56	0.32	3.50	11.44	73.36	1.34
SWS 4 (50 < d < 75 µm)	1.60	7.79	0.56	0.32	3.36	10.67	74.29	2.17
SWS 5 (50 < d < 75 µm)	1.87	7.77	0.72	0.32	3.32	10.77	73.54	2.34
SWS 6 (50 < d < 75 µm)	1.87	7.72	0.73	0.30	3.26	10.69	73.05	2.35
Average	1.74	7.73	0.68	0.32	3.47	10.89	73.57	1.93
Standard deviation	0.13	0.31	0.12	0.01	0.22	0.44	0.53	0.52

**Table 6:** Normalised Reference clinker main XRD phase composition (wt%).

	1250 °C	1350 °C	1450 °C	Typical Range
Hatrurite (C_3_S)	4.47	13.74	38.54	50–70
Larnite (β-C_2_S)	13.77	5.45	21.79	15–30
Calcio-olivine (γ-C_2_S)	5.37	69.67	29.11	NA
Brownmillerite (C_4_AF)	12.34	3.44	2.93	±10
Tricalcium aluminate (C_3_A)	NA	NA	NA	5–10
Portlandite (Ca(OH)_2_)	40.43	0.47	0.65	NA
Lime (CaO_f_)	15.74	4.98	5.37	<2
Periclase (MgO_f_)	7.87	2.25	1.63	<5
Total	100.00	100.00	100.00	

NA—the phase was not detected.

**Table 7:** Normalised SWS clinker main phase composition (wt%) values for the peak temperatures.

	1250 °C	1300 °C	1350 °C	1450 °C	Typical Range
Hatrurite (C_3_S)	11.96	29.00	55.64 ± 2.26	40.72	50–70
Larnite (β-C_2_S)	28.04	23.59	18.67 ± 1.27	25.90	15–30
Calcio-olivine (γ-C_2_S)	17.76	15.37	15.53 ± 3.11	26.22	NA
Brownmillerite (C_4_AF)	12.52	19.70	4.62 ± 1.56	1.95	±10
Tricalcium aluminate (C_3_A)	NA	NA	NA	NA	5–10
Portlandite (Ca(OH)_2_)	13.27	0.65	0.28 ± 0.07	1.47	NA
Lime (CaO_f_)	8.41	4.33	1.57 ± 0.35	1.79	<2
Periclase (MgO_f_)	8.04	7.36	3.70 ± 1.41	1.95	<5
Total	100.00	100.00	100.00	100.00	

NA—the phase was not detected.

**Table 8:** Reference and SWS clinker amorphous phase content (wt%) at peak temperatures.

	1250 °C	1300 °C	1350 °C	1450 °C
Reference Clinker	37.6	-	13.3	37.2
SWS Clinker	45.5	53.4	45.0	36.6

## Data Availability

The original contributions presented in this study are included in the article. Further inquiries can be directed to the corresponding author.
